# A pan-cancer, pan-treatment model for predicting drug responses from patient-derived xenografts

**DOI:** 10.1093/nargab/lqaf111

**Published:** 2025-08-28

**Authors:** Shruti Gupta, Vikash K Mohani, Ghita Ghislat, Pedro J Ballester, Shandar Ahmad

**Affiliations:** School of Computational and Integrative Sciences, Jawaharlal Nehru University, New Delhi, India; School of Computational and Integrative Sciences, Jawaharlal Nehru University, New Delhi, India; The Francis Crick Institute, London NW1 1AT, UK; Department of Bioengineering, Imperial College London, London SW7 2AZ, UK; Centre de Recherche en Cancérologie de Marseille (CRCM), INSERM, U1068, F-13009 Marseille, France; School of Computational and Integrative Sciences, Jawaharlal Nehru University, New Delhi, India

## Abstract

The translatability of patient-derived xenograft (PDX)-generated clinical data into patient-specific outcomes for therapeutic guidance is limited by the challenges in generalizability of models across patients, treatments, and cancer types. Previously, machine learning (ML) models have been developed for the two most abundant cancer types, i.e. breast cancer and colorectal cancer, but these are unusable in other cancer types because each treatment/cancer type requires a different model to be trained. Here, we provide an ML framework to train a single pan-cancer, pan-treatment model for predicting treatment outcomes. We show that such models give promising results for all cancer types considered and reproduce the accuracy levels of individually trained cancer types. In the proposed model, all PDX genomic profiles from all cancer types are used as the training data, and instead of partitioning them into cancer types for each model, the cancer type and treatment name are appended as the input features of the training model. Using genomic-only and treatment-only embeddings and combining them with principal component analysis-based dimensionality reduction, our models show promising results and provide a framework for further improvements and real-time use for best treatment selections for cancer patients.

## Introduction

Despite significant progress in cancer therapeutics, the prognosis remains poor and highly unpredictable [1–3]. It has now become clear that the cancer state and general profile of every patient determine if and to which drug they will respond effectively [4–6]. Some basic profiling of patients into their genetic groups and cancer types has improved clinical outcomes, but the goal of patient-specific treatment selection remains far from realized [4, 7].

Accurate *a priori* prediction of outcomes for each possible treatment option requires experimental data that represent the specific patient's tumor and other conditions, together with drug responses and the development of a computational model through biomarkers or whole profile methods to predict patient outcomes in real-time clinical conditions [8]. Traditional cell line experiments, ADMET (absorption, distribution, metabolism, excretion, and toxicity) studies, and animal pharmacological models have been used to test drug response under different scenarios. However, animal and cell line models do not effectively represent the tumor environment or patient's profile due to differences in their genomic, mutational, and transcriptomic profiles [9].

The need for developing drug response experimental models most closely representing real individual patients has led to several novel systems, of which patient-derived xenografts (PDXs) are among the most powerful [10–12]. PDX technology allows the transfer of cancer tissue onto a number of immunocompromised mice, where it is grown as a patient's experimental model. The potential effect on patients is tested by treating individual mice with the corresponding treatment and recording their responses. PDX models are known to have the most remarkable similarity to human diseases yet achieved. In this way, the patient's response to multiple treatments can be tested, which is impossible for real patients. There are many noted examples where PDX models have been used as guides for treating patients [13–19]. PDXs maintain the genetic and epigenetic mutations and other features that contribute to drug resistance, and their response to drugs is correlated with those observed in the clinic [20–25].

PDX models are accurate but time-consuming and, therefore, not suitable for a real-time selection of the best treatment. However, data generated from controlled experiments on PDX can be used to learn about the relationship between the genotype and transcriptome profile of a tumor on the one hand and the differential response to different therapeutic options on the other hand, e.g. predicting if a patient with a given genomic and transcriptomic profile would be resistant or sensitive to a specific treatment. In the past, machine learning (ML) and deep learning (DL) algorithms have been trained on high-throughput data to develop models that can predict the response of cancer cell lines to novel drugs and drug combinations [26]. Among the many ML algorithms used in drug response prediction, linear regression, support vector machines (SVMs), ridge regression, lasso regression, elastic net, naive Bayes, neural networks, deep neural networks (DNNs), random forest (RF), gradient boosting machines (GBMs), k-means, hierarchical clustering, and decision trees [27–33] have also been used. Decision trees represent events/decisions as nodes/branches/endpoints and are easily interpretable and computationally efficient. The RF model relies on developing an ensemble of decision trees, and this has performed well on many biological problems.

An RF-based model on PDX datasets has been developed by Nguyen *et al.* [34] to predict the drug response for each treatment and cancer type pair on the NIBR-PDXE dataset (Novartis Institutes for Biomedical Research PDX Encyclopedia) [35]. The model led to the most accurate predictions when the most predictive features were used and selected through a criterion of optimal model complexity (OMC), a strategy developed by the authors to build ML models using the most relevant features. They also showed that multiple gene classifiers had higher recall *in vivo*, consistent with *in vitro*-based similar studies. They identified the most suitable profile, developed a classifier for each treatment, and assessed which molecular profile is most predictive for a given drug. One of the limitations of the models used by Nguyen *et al.* [34] was that the number of PDX experiments available for each cancer–treatment pair was small, and hence, for a large number of them, no model could be developed.

In this work, we propose a novel solution to the problem of insufficient data in developing PDX-based predictive models by introducing a framework that pools training data from all cancer types and treatments. Ordinarily, integrating multiple datasets for different classes of cancer and treatment leads to multi-class problems that do not overcome sparsely populated classes in the data because the training samples in each class remain insufficient. Our approach is to solve this problem by treating the cancer types and treatments as part of the input features instead of target classes, thereby developing a single binary class model with a larger number of training instances. We believe this approach will allow us to train models to capture interactions between similar genomic features across cancer types and intrinsically determine their contributions specific to cancer and treatment types. This so-called pan-cancer pan-treatment (PCPT) model uses a unified feature representation set by appending the cancer types and treatment types to transcriptomic and other personalized input features. To overcome the high dimensionality of the input dataset, we used principal component analysis (PCA) for feature extraction. We observed that the integrated PCPT model could be trained with promising accuracy levels across cancer and treatment types, and reproduce the performance of the models with sufficient datasets reported earlier with comparable levels [34]. Although developed for PDX datasets, the proposed approach is intuitively scalable to other multi-class problems with sparse datasets.

## Materials and methods

**Figure 1.**
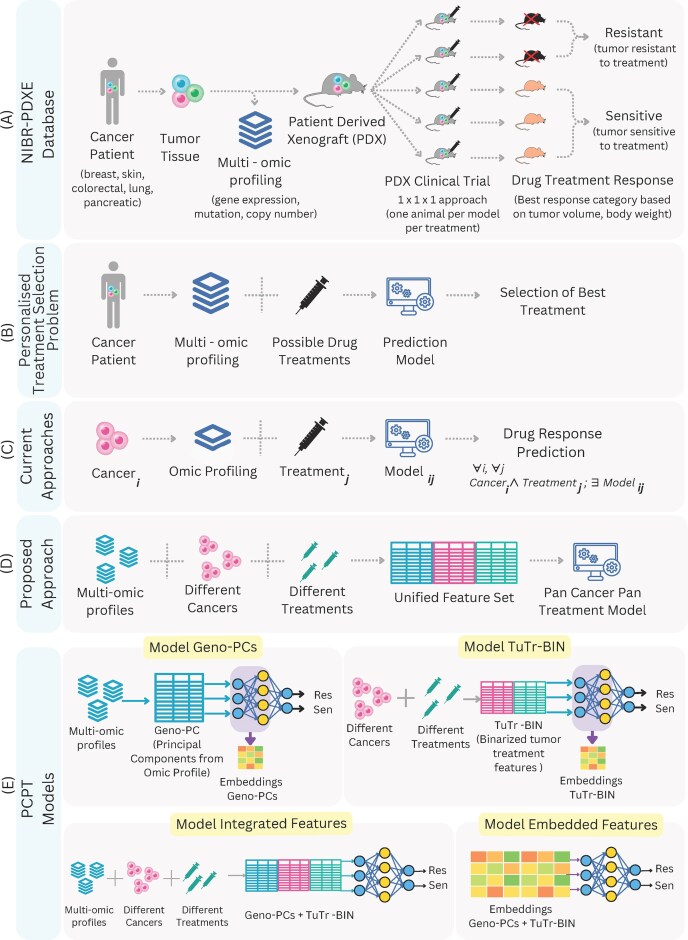
(**A**) Overview of NIBR-PDXE dataset generation. (**B**) Personalized treatment selection problem. (**C**) Current approaches. (**D**) Proposed PCPT approach. (**E**) Representation of different PCPT models..

The overall aim of the PDX-based predictive models is to characterize a patient-specific tumor in terms of its genomic and related features. Then, for each treatment tested, it computationally predicts the outcome, i.e. whether the patient is sensitive or resistant to the corresponding treatment. Specifically, the problem to be addressed here is to take the genomic features of a PDX, including gene expression profiles, copy number variation, and mutations observed, and then give a treatment and the cancer type to which a PDX sample belongs, and predict if this treatment will be effective (PDX responsive) or not (PDX non-responsive). The overall strategy of our model is two-fold. First, the high-dimensional feature data on the genomic profiling of each PDX are condensed by a PCA-based approach. Secondly, instead of training these reduced features on each cancer type and for each treatment for each PDX, a cumulative model is developed in which cancer and treatment are fed as features instead of a target class (see Fig. 1). This allows a single model to be applied to any cancer type and treatment used in this cumulative model. A detailed description of the datasets, pre-processing, and training steps used are described below.

### Ethics statement

We declare that the current study is based on publicly available data with no human subjects involved, and therefore no ethical clearance is needed. All computational work follows ethical standards of reporting.

### Dataset description

**Figure 2.**
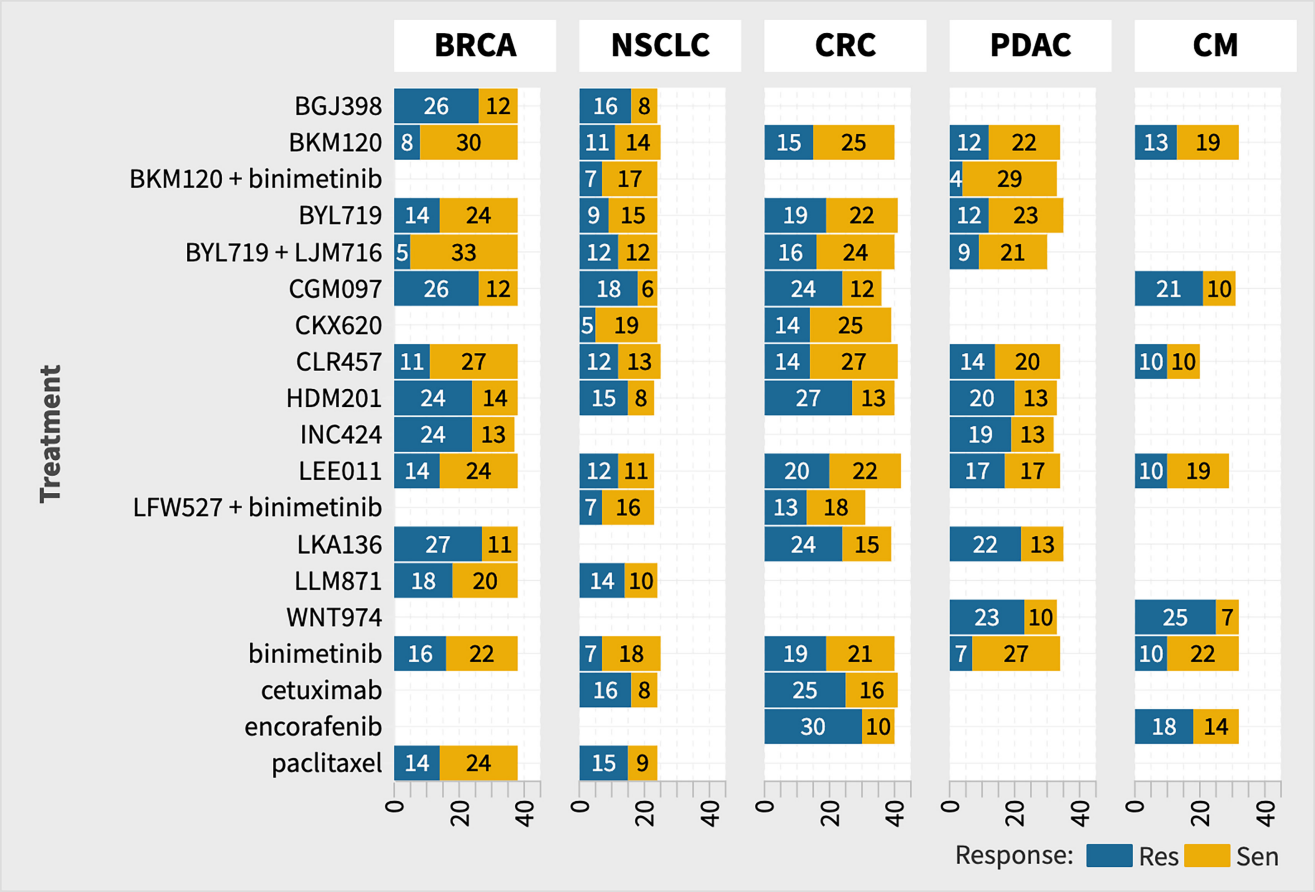
Number of samples tested for each treatment–cancer pair grouped by their response to treatment.

We used the NIBR-PDXE dataset. The dataset contains ∼400 PDX models of different tumor types characterized by their mutation, copy-number alterations, and mRNA expression information, and their response to drug treatments measured in terms of tumor size. The dataset studies five tumor PDX models, namely BRCA (breast carcinoma), CM (cutaneous melanoma), CRC (colorectal cancer), NSCLC (non-small cell lung carcinoma), and PDAC (pancreatic ductal carcinoma). The different treatments applied on these PDXs are BGJ398, binimetinib, BKM120, BYL719, BYL719 + LJM716, CGM097, CLR457, HDM201, INC424, LEE011, LKA136, LLM871, paclitaxel, encorafenib, WNT974, cetuximab, CKX620, LFW527 + binimetinib, and BKM120 + binimetinib. The final dataset used in this study is summarized in Table 1, and a detailed sample count for each combination is provided in Fig. 2. These statistics are derived from the original data available as an xlsx file containing five sheets named RNASeq_fpkm, copy_number, pdxe_mut_and_cn2, PCT_raw_data, and PCT_curve_metrics which contains gene expression data, actual gene copy number, mutations, and categorical copy number alterations, raw response in terms of tumor volume change, and the processed response of PDX into different classes, respectively, in previous works, where additional details of datasets are available [34, 35]. These data are referred to as Gao data in the rest of this work.

**Table 1.** Summary of the experimental data used in this study

Details of the processed dataset	Counts
Number of cancers	5
Number of treatments	19
Number of PDXs	186
Types of genomic geatures	4
Genomic features	72545
GEX	21107
CNV	23852
CAN	14220
SNV	13366
Total combinations of treatment–cancer type–genomic feature	1938

### Data pre-processing

The Gao dataset generated five different genomic information matrices to describe individual tumors, namely gene expression matrix (GEX), copy number variation (CNV), copy number alterations (CNA), single nucleotide variation (SNV), and PDX clinical trial (PCT) [34, 35] (see Table 1). They were pre-processed or used as follows.

- The GEX matrix in the RNASeq_fpkm sheet contains normalized gene expression values in FPKM (fragments per kilobase of transcript per million mapped reads). It contained expression data for 22665 genes across 399 PDX samples and was used without additional pre-processing.
- The CNV matrix in the copy_number sheet contains the copy number of 23852 genes across 375 PDXs. The data were directly used as provided in the sheet.
- The CNA matrix was derived from the pdxe_mut_and_cn2 sheet, where categorical copy number data (e.g. Amp5, Amp8, Del0.8) were binarized. Aberrant copy numbers were marked as 1, and normal values as 0. The final matrix contains 21818 features for 399 PDXs. The data were directly used as provided in the sheet.
- The SNV matrix is derived from the pdxe_mut_and_cn2 sheet and was generated by binarizing the mutation data. A value of 1 indicates the presence of at least one somatic mutation in a gene, while 0 indicates no mutation. The final matrix contains 21818 binary features for 399 PDXs.
- The PCT matrix is extracted from the PCT_raw_data and PCT_curve_metrics sheets, and includes recalculated response categories based on Nguyen *et al.* [34] namely Sen (sensitive or respondent) and Res (resistant or non-respondent).

After combining the genomic feature matrices and merging them with the treatment profiles for each PDX model, we removed columns with missing data. This resulted in 186 unique PDX models and 1938 tumor–treatment pair combinations. The final dataset consists of 1938 data entries, representing combinations of treatment, cancer type, and genomic features across five cancer types. Only PDX models with complete molecular profiling and response data for at least three samples in the respondent or non-respondent categories were retained.

### Feature engineering

#### Genomic feature extraction using prinipal component analysis

We applied PCA using the PCA module from the scikit-learn library to reduce the dimensionality of the genomic data, which initially consisted of 72545 features (see Table 1). We selected the top 200 principal components (PCs) as representative of all these features (see the Results). Before performing PCA, the genomic features were standardized in Python by subtracting the mean and scaling to unit variance using the StandardScaler function from the scikit-learn library [36]. The resulting set of 200 PCs, referred to as “Geno-PC” throughout the manuscipt, was used as the input feature set for subsequent analyses.

#### Tumor and treatment class features

To develop the PCPT model, we included categorical features representing both tumor types and treatment types. These were one-hot encoded, resulting in 24 binary features: 19 for treatment types and 5 for tumor types. This set of 24 binary features is referred to as “TuTr-BIN” in the manuscript and is appended as an input vector (resulting in 224 features for each sample) for our classifier model.

### Pan-cancer, pan-treatment model and training strategy

As outlined above, we adopted a novel strategy wherein we treated cancer and treatment types as input features and developed a unified feature representation set. This contrasts with previous studies, where a different model is trained for each combination. The predictive model was developed on PCPT representation to classify patients into respondent and non-respondent classes.

We used a simple feed-forward neural network consisting of one input layer, a hidden layer with eight nodes, and an output layer. In our study, we used either the rectified linear unit (ReLU) or the tanh activation function in the hidden layer and applied a sigmoid activation function in the output layer to train a binary classification model.

To optimize the network's trainable parameters (weights and biases), we used the binary cross-entropy loss function to measure the difference between predicted probabilities and true binary labels. We applied a dropout rate of 0.2 in the hidden layer to prevent overfitting. Dropout helps by randomly deactivating neurons during training, effectively averaging multiple models and reducing overfitting [37]. We initialized the weights using Glorot normal distribution sampling [38]. We trained the model using the Adam optimizer with a fixed learning rate of 1.00E-04. The network was trained for 500 epochs with a batch size of 64.

We explored different representation strategies of the features and trained models for each representation. These models are described in Table 2. Unless otherwise noted, all models were trained with the same parameters. This structured approach allowed us to test and compare various feature sets, leading to the development of the final PCPT model that combines predictions from the previous models for enhanced performance. Apart from the original 200 PCA features and 24 features, which included the tumor and treatment-derived features, additional approaches based on auto-encoder-like embeddings were also used, as listed in Table 2 and described below.

**Table 2.** Summary of novel models, feature representation strategies, and hyper-parameters

Model	Input feature set description	Input size (features × samples)	Activation function
Model-Geno-PC	Geno-PC feature set	200 × 1938	ReLu
Model-TuTr-BIN	TuTr-BIN feature set	24 × 1938	tanh
Model-Integrated-features	Combined Geno-PC and TuTr-BIN feature sets	224 × 1938	ReLu
Model-Embedded-feature	Embeddings from Geno-PC and TuTr-Bin models	16 × 1938	tanh
Model-PCPT	Average of the prediction scores of the above four models	4 × 1938	–

The number of hidden layers in each model is one with eight nodes. Activation and loss of function in each model are sigmoid and binary cross-entropy.

### Embedding extraction process

In our model development, we created a Model-Embedded-Feature by extracting embeddings from the Model-Geno-PC and Model-TuTr-BIN, the response class predictions trained exclusively from genomic PCA (200 features) and tumor–treatment (24 features), respectively. The embeddings represent compressed, lower dimensional representations of the original feature sets, allowing us to efficiently integrate the information from genomic features and tumor–treatment classifications.

For the Model-Geno-PC, we trained a neural network on the Geno-PC, and the activations from the hidden layer were used as the embeddings. Similarly, for the Model-TuTr-BIN, the activations from the hidden layer were extracted as embeddings representing tumor and treatment type features (TuTr-BIN).

These embeddings were then concatenated, resulting in a combined embedding of size 16 for each sample. This embedded representation was used as input for the Model-Embedded-Feature, where the compressed information from genomic and class features, together with cross-validation-based training, allowed for efficient classification.

### Model training and cross-validation

We employed 4-fold cross-validation to evaluate model performance, using a proportional sampling approach for tumor–treatment pairs. Samples from each tumor–treatment pair were distributed proportionally across the folds. Pairs with fewer samples contributed fewer instances per fold, while pairs with more samples contributed more. This ensured that each fold maintained a proportional representation of the tumor–treatment pairs, preventing any pair from being over- or under-represented during training and evaluation.

During each fold training and testing, we maintained a strict separation between training and test data, preventing information leakage and ensuring accurate model performance estimates, e.g. by computing embeddings for each iteration only from the models based on training data, ensuring the integrity of the evaluation process and helping us to estimate true performance levels on unseen samples.

### Evaluation of the performance of different pan-cancer, pan-treatment models

We evaluated the models using key metrics, including the F1 score and Matthews correlation coefficient (MCC), chosen for their robustness in handling imbalanced classes. The decision threshold was optimized based on the true-positive rate (TPR) and false-positive rate (FPR). Performance was assessed globally across all tumor–treatment pairs, individual tumor types, and treatments to identify any model biases and ensure broad generalizability.

## Results

As described in the Materials and methods, we have tested multiple methods of creating an integrative prediction to arrive at treatment response status from genomic feature sets (Table 2). In each model, our starting representation of genomic features is the cross-validated PCs (transformation matrices derived only from the training data). In this section, we systematically describe the results and analysis of the training models.

### Principal components of genomic features of patient-derived xenografts

**Figure 3.**
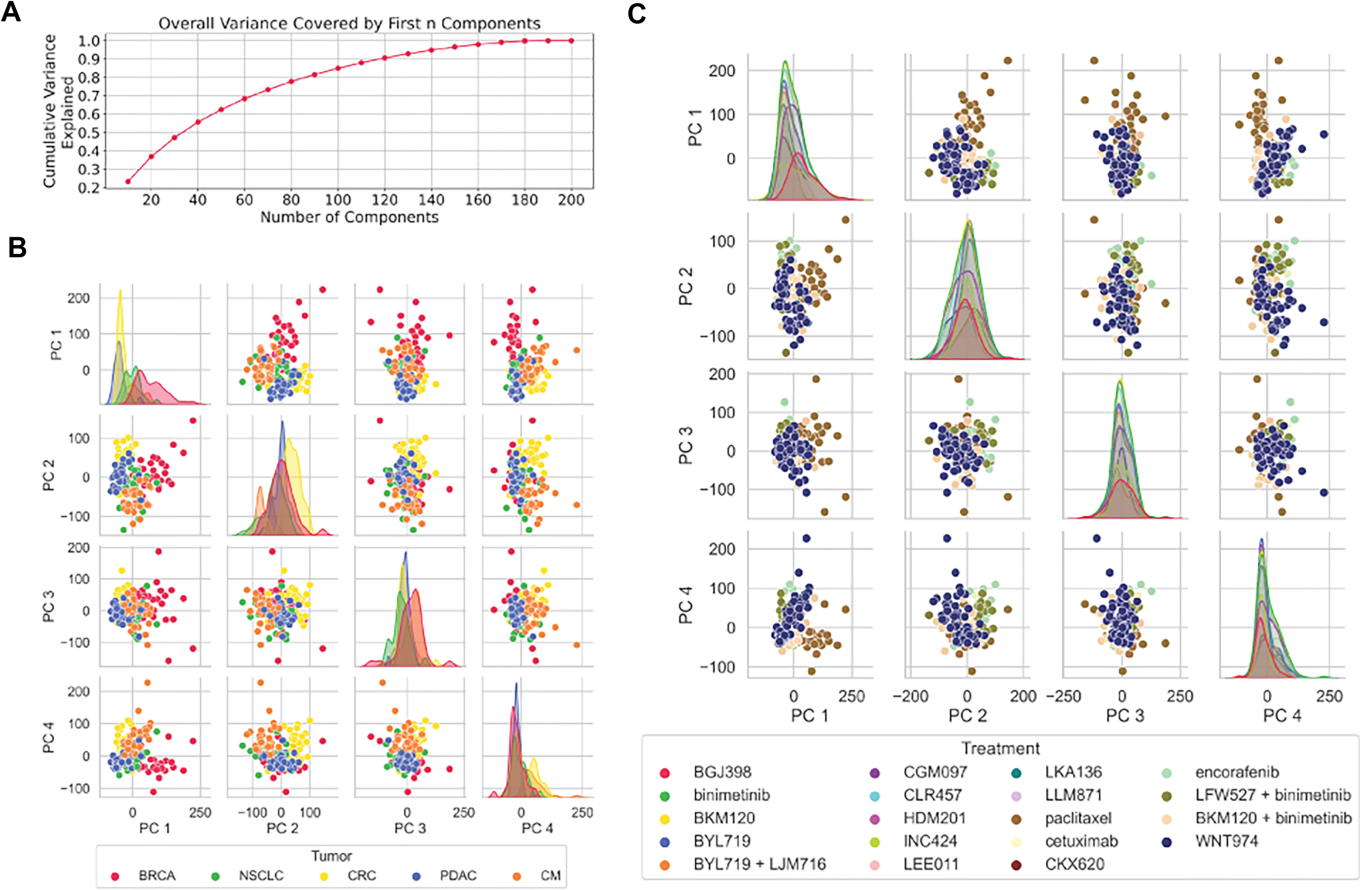
Principal component analysis (PCA) of genomic features in all PDX samples. (**A**) Variance explained by different numbers of top principal components. (**B**) Separation of different cancer types in the top four components of PCA space. (**C**) Distribution of samples given different treatments across all cancer types in the top four components of PCA space.

We extracted PCs from 72 545 features covering the four molecular profiles of the samples. First, we examine all the data to explore the true dimensionality of genomic features. Figure 3A shows the scree plot representing the cumulative variance captured by increasing the number of selected components. A total of 10, 20, 50, 100, and 150 PCs explain 23.23, 36.83, 62.51, 84.94, and 96.56%, respectively. The top 200 components cover most of the variance in the data and form the feature representation of our genomic information for subsequent predictive models.

The PCA of genomic features by pooling all the cancer types and treatments presents us with the opportunity to explore cancer-specific variations in PC space. These individual variations are critical to differential responses to each treatment and transfer of training across cancer types at the feature representation level. Different tumor types are expected to have some genomic profiles specific to cancer type, irrespective of their response to treatment.

However, treatments are an external intervention and, therefore, are blind to genomic PCs. Any distinction in PCs in treatment space may simply mean the differences in the choice made of patients to receive that treatment. They may also represent random variations in genomic profiles captured as biases due to a small number of samples in some treatments. In Fig. 3B, we observe a clear, narrow peak in density plots of PC1 and PC2 for CRC and PDAC, respectively, which suggests less variability within the cancer type concerning the molecular features captured by that component. In addition, we observe that PC1 can separate the BRCA cancer types well, and PC4 also shows a clear, narrow peak for BRCA. A separation of CRC and CM in PC2–PC4 is also observed. Our treatment response model aims to distinguish between tumor responses and not the tumor itself, although the intrinsic differences in tumor profiles are inevitable. This analysis shows that the treatment-independent separation of cancer types within the PCs is a major challenge that any predictive model must overcome in effectively learning a treatment-specific response across cancer types. Figure 3C confirms that the treatments are generally not separated much between the top PCs and, therefore, meet an important prerequisite for the data quality and consistency of the proposed models. However, we observe that paclitaxel is somewhat separated from the rest of the clusters in the scatter plot, although there is no distinct peak in density distributions. This suggests that the nature of patients given this treatment was slightly biased, irrespective of their response levels to treatments. This bias is observed only in one treatment; therefore, we have made no attempt to correct it in the current work.

### Prediction performance of the best pan-cancer, pan-treatment model

**Figure 4.**
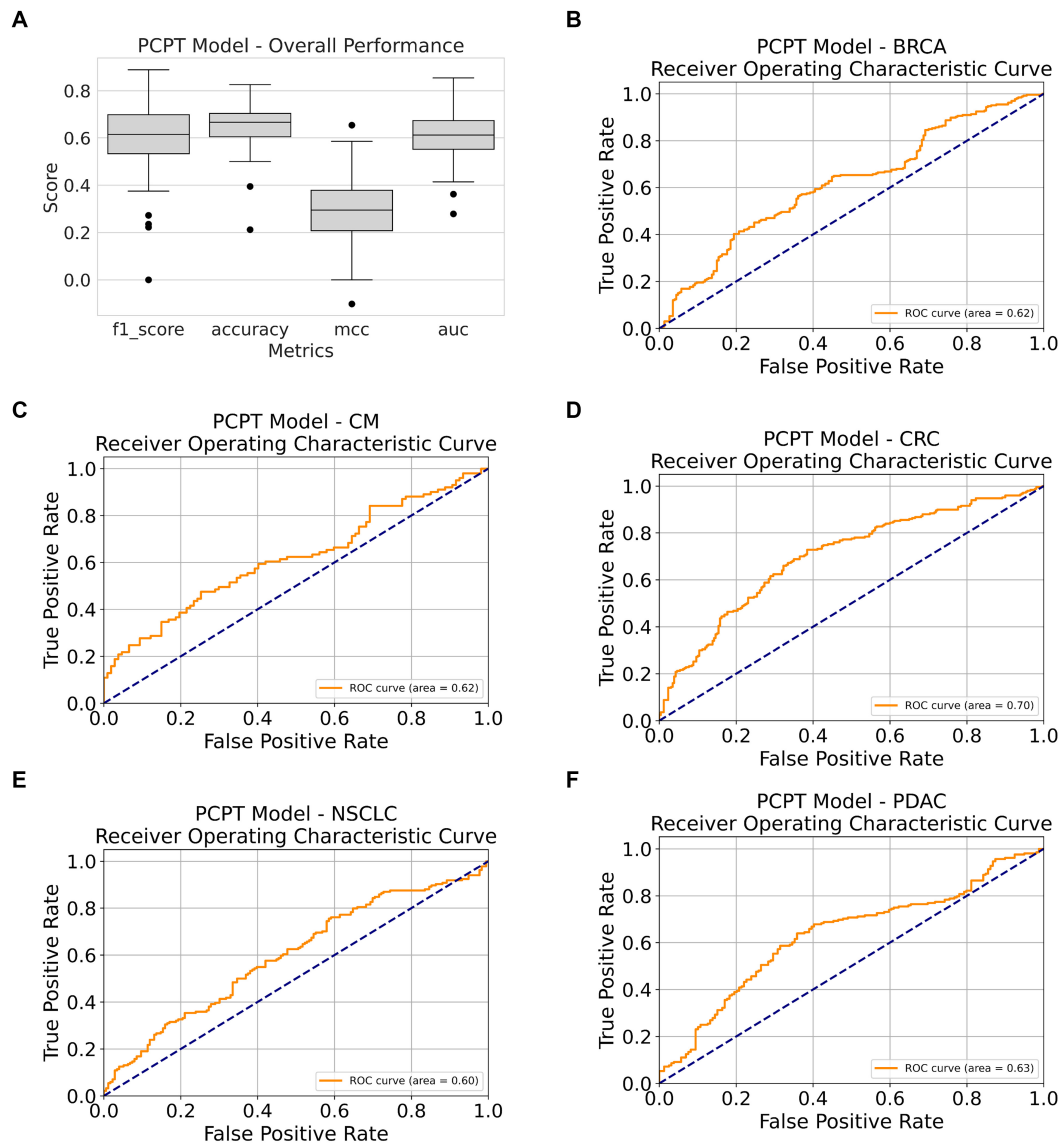
Predictive performance of the PCPT model. (**A**) Overall performance scores for each tumor–treatment pair measured by different metrics. (**B–F**) The area under the receiver operating characteristic curve for each tumor type.

As shown in Table 2, we have used five different types of PCPT models. We discuss the outcomes of each of these models in a later section. We first discuss the performance levels of the best PCPT model (simply called the PCPT model) in our work, and it is based on the predicted scores for each PDX sample obtained from four different trained models. It may be noted that all prediction scores are based on the predictions for the samples when they were in the test datasets and represent the true generalizability of the model under a cross-validation scheme, preventing information leakage from the training steps. Figure 4A shows that the final PCPT model performance varies somewhat across tumor–treatment pairs. While the F1 scores, accuracy, and area under the curve (AUC) indicate a reasonable level of performance, the low MCC values could be due to class imbalances. It should be noted that for highly class-imbalanced data, MCC scores often underestimate predictive power, and thus the other scores must be considered as the guide [39]. Figure 4B–F shows that the receiver operating characteristic (ROC) curve of the PCPT model for BRCA is 0.62, for CM is 0.62, for CRC is 0.70, for NSCLC is 0.60, and for PDAC is 0.63. Prima facie, a performance range between 0.62 and 0.70 observed here may look modest, but these results must be seen in the following context. First of all, for some of the tumor–treatment types, the number of samples is too small, and it has so far not been possible to train a model exclusively on such data. Thus, whatever prediction performance we obtain here is good because these models have been trained using ML for the first time and, to date, is the only way tried. Secondly, in a high-dimensional system such as this, overfitting is a great risk, and high performance levels are often misleading because generalization is low. Current models have been trained with strict cross-validation controls and represent a high confidence of generalization. Third, the genomic profiles are not the only factor that determines the effectiveness of a treatment, as there are many confounding factors, such as non-genomic factors, individual host variation, and cellular heterogeneity. In the current model, genomic features alone have been shown to provide us with useful leads for treatment selection, which can be combined with other clinical leads to provide a more powerful personal therapy. Finally, the field of PDX-derived ML models for treatment response is still in an early stage, and future models will improve the performance. In contrast, the proposed model provides a framework for working with a large number of classes with a low number of samples in each group.

**Figure 5.**
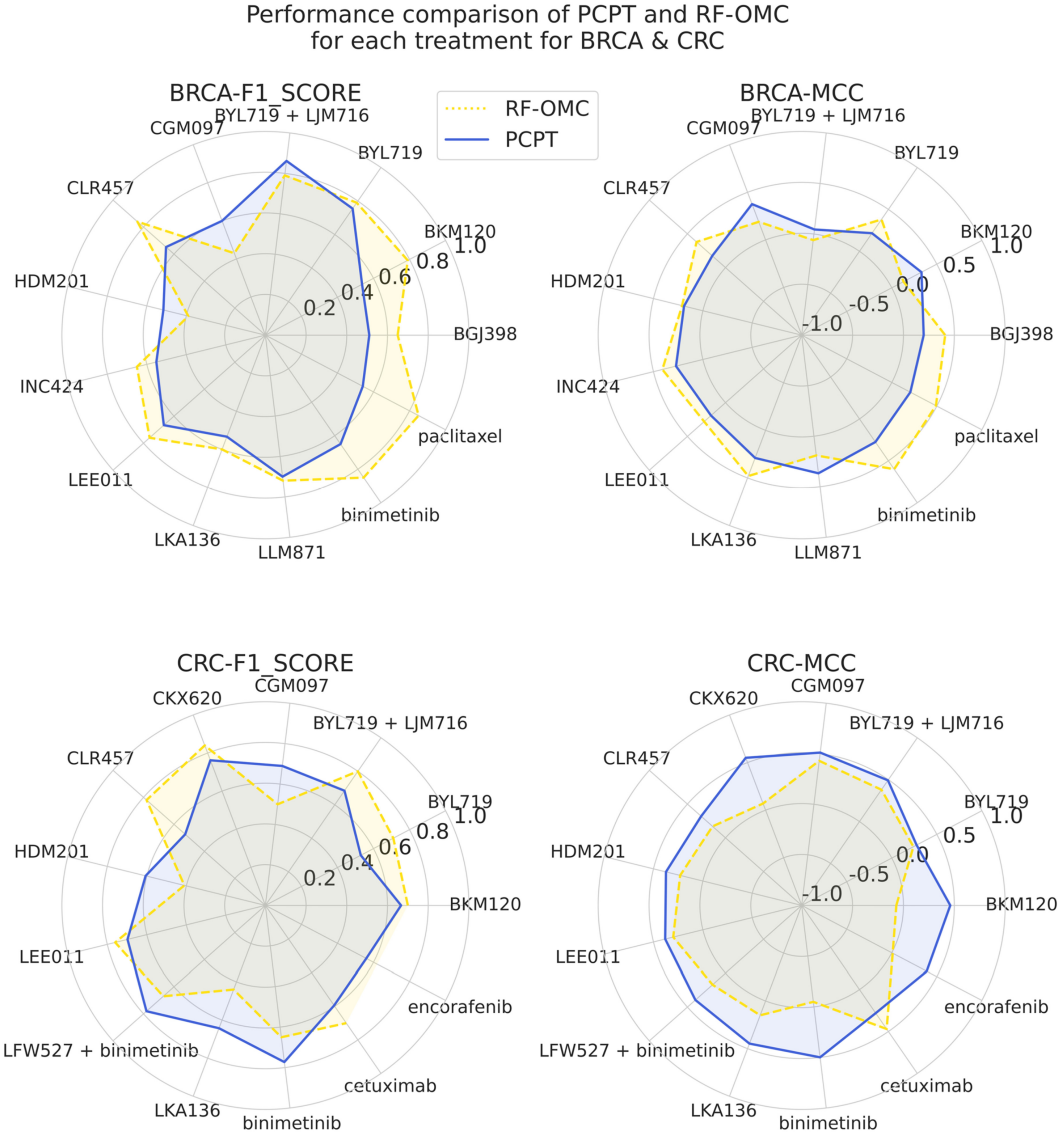
Comparison of the PCPT model with the RF-OMC model for BRCA1 and CRC for all treatments.

As stated above, many of the tumor–treatment pairs are trained here for the first time and cannot be compared with anything in the literature. However, BRCA and CRC systems have sufficient data, and effective models have been developed for them previously. We compare in the following if the PCPT model trained in combination with all cancers can retain the performance on samples of these two cancers reported by the previous RF-OMC model proposed by Nguyen *et al.* [34] We also compare the performance levels of PCPT precursor models (first four rows of Table 2) with the finally aggregated PCPT model for these and other cancer types in the subsequent sections. Figure 5 shows the comparison of the PCPT model with the RF-OMC model of Nguyen *et al.* [34] on BRCA and CRC tumors. Since only the F1 score and MCC have been reported in previous work, the comparison is made only for these scores. We observe that for some treatments, PCPT outperformed the RF-OMC model, and vice versa in other cases, and that the prediction performance levels from the two models are comparable (see Table 3). Specifically, BRCA results are better for RF-OMC models, whereas PCPT outperforms for CRC, making it overall a negligible difference. In the case of BRCA, an overall drop in performance is actually driven by three treatments, most prominently paclitaxel (see Fig. 5), which was also flagged for its special behavior in the PCA in the section above. It appears that the genomic profile data belonging to some treatments have a bias that is difficult to capture in a larger model relying on global deterministic signatures of treatment response. An apparent anomaly in the PDX data for some of the treatments, especially paclitaxel, suggested here is a matter of further investigation in the future. Here, we conclude that the overall prediction performance levels suggest that even for the cancer types with sufficient data, PCPT could reproduce at least the same accuracy levels obtained by specialized models trained on only one cancer type. This result may be used to gain further confidence in the prediction performance of cancer types never trained using ML models so far.

**Table 3.** Comparison of prediction performance between PCPT and RF-OMC models for the cancer types trained previously as individual models (BRCA and CRC)

*(A) Averall performance for common cancer types in the two models*						
	F1 score			MCC		
Cancer type	RF-OMC (mean)	PCPT (mean)	*P*-value	RF-OMC (mean)	PCPT (mean)	*P*-value
BRCA	0.70	0.6211	0.0681	0.31	0.2408	0.1752
CRC	0.66	0.6627	0.9894	0.19	0.4133	0.0032
***(B) Treatment-wise comparison of performance***						
**Tumor**	**Treatment**	**PCPT (F1**)	**RF-OMC (F1**)	**PCPT (MCC**)	**RF-OMC (MCC**)	
BRCA	BGJ398	0.5106	0.65	0.1989	0.41	
BRCA	Binimetinib	0.6500	0.85	0.2753	0.60	
BRCA	BKM120	0.5366	0.79	0.3296	0.13	
BRCA	BYL719	0.7547	0.79	0.2161	0.38	
BRCA	BYL719 + LJM716	0.8615	0.79	0.0449	–0.06	
BRCA	CGM097	0.6000	0.43	0.3760	0.19	
BRCA	CLR457	0.6522	0.84	0.1741	0.38	
BRCA	HDM201	0.5161	0.39	0.1906	0.21	
BRCA	INC424	0.5517	0.65	0.2718	0.41	
BRCA	LEE011	0.6667	0.76	0.1906	0.28	
BRCA	LKA136	0.5333	0.60	0.2901	0.48	
BRCA	LLM871	0.7000	0.72	0.3667	0.19	
BRCA	Paclitaxel	0.5405	0.85	0.2058	0.49	
CRC	Binimetinib	0.7727	0.65	0.4988	–0.05	
CRC	BKM120	0.6667	0.70	0.4601	–0.07	
CRC	BYL719	0.5294	0.71	0.2753	0.24	
CRC	BYL719 + LJM716	0.6842	0.80	0.4922	0.38	
CRC	Cetuximab	0.5946	0.70	0.2806	0.47	
CRC	CGM097	0.6897	0.50	0.5115	0.43	
CRC	CKX620	0.7619	0.84	0.5500	0.07	
CRC	CLR457	0.5263	0.78	0.3199	0.17	
CRC	encorafenib	0.5600	NA	0.3876	0.01	
CRC	HDM201	0.6061	0.41	0.3736	0.23	
CRC	LEE011	0.6977	0.76	0.3814	0.30	
CRC	LFW527 + binimetinib	0.7805	0.67	0.3952	0.17	
CRC	LKA136	0.6429	0.44	0.4472	0.15	

### Performance comparison of different pan-cancer, pan-treatment models

**Figure 6.**
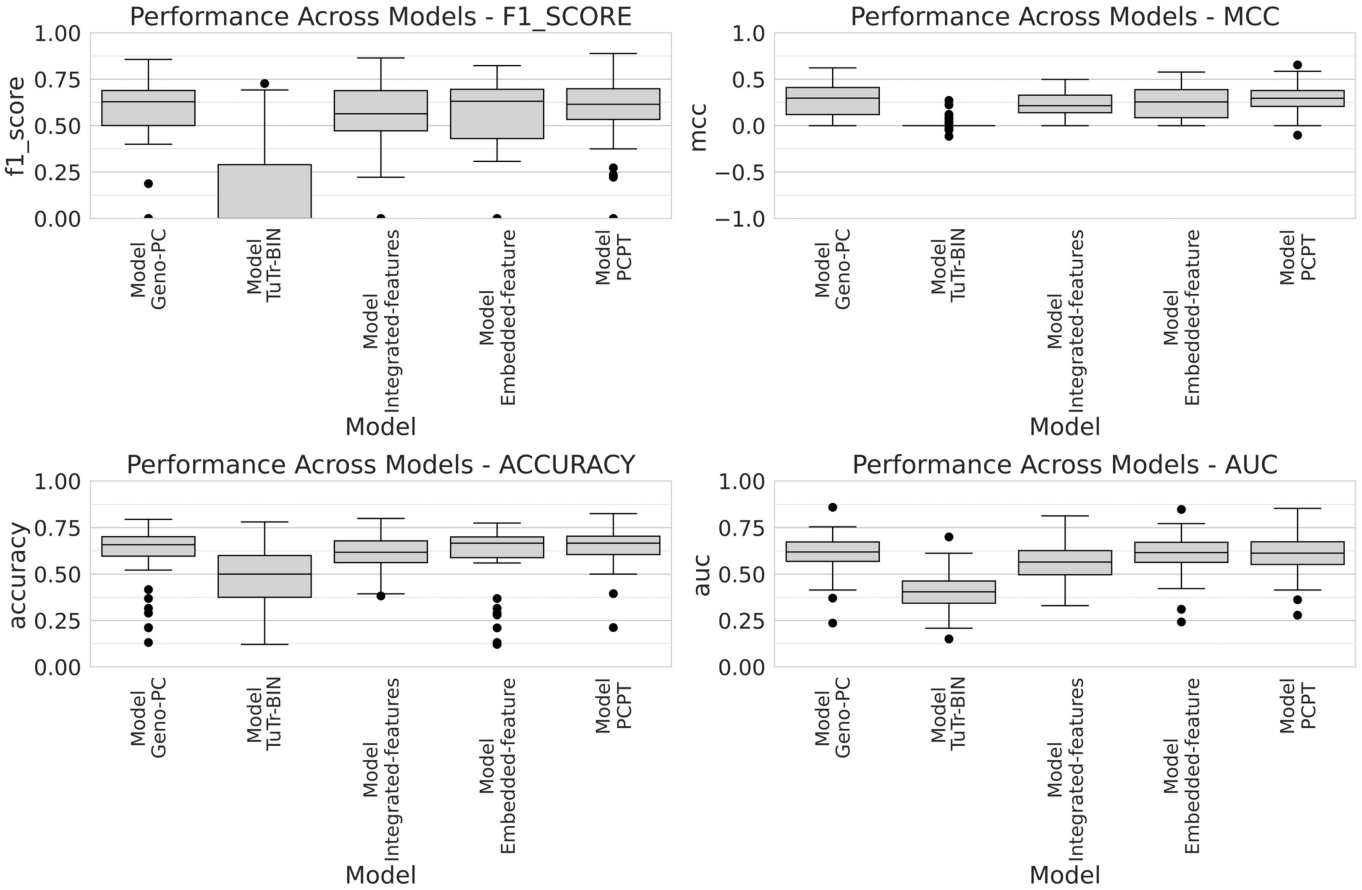
Overall performance of different PCPT models for all tumor–treatment combinations.

As described in the Materials and methods, we used four different predictive models by representing genomic information of PDX samples in different ways, and the final PCPT model averages the predictions obtained from each of the four models. Here, we examine the predictive power of each of the four constituent precursor models to estimate the nature of our models' predictive information. Figure 6 shows the treatment-wise distribution of prediction performance of different PCPT models in terms of four performance metrics. We observe that the models based on the PCA representation of genomic profiles and their corresponding low-dimensional embeddings serve well and contribute maximally to the final PCPT models. Treatment alone is not predictive, which indicates that the model has effectively eliminated baseline effectiveness levels of drugs. Nonetheless, the final PCPT model is slightly better than all the precursor models and more robust as the interquartile ranges are somewhat lower than the genomics-only features.

### Class imbalance and prediction performance

**Figure 7.**
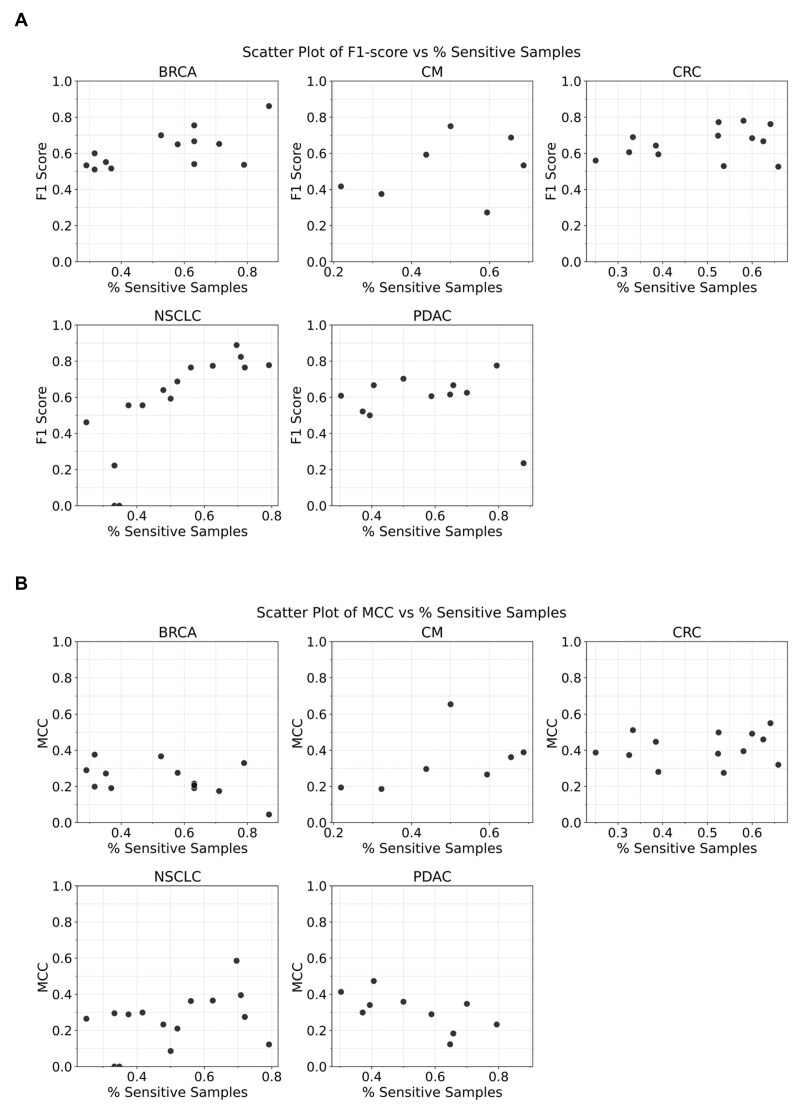
Cancer-wise trends on prediction performance trends with respect to the size of treatment data in the sensitive group (class imbalance).

We observe that the number of sensitive and resistant samples for each treatment and cancer type is not proportionate in the datasets (see Fig. 2). This class imbalance may not only impact the trainability of models but may also produce misleading prediction scores, e.g. MCC has been reported to underestimate performance for class-imbalanced datasets. In Fig. 7, we show the model F1 and MCC scores as a function of the relative number of sensitive data for the corresponding tumor–treatment pair. An overall look at these plots suggests that only the NSCLC models show a significant correlation between the F1 score and the number of samples in the sensitive class. From observations made from the five cancer types and only in the F1 score, it is difficult to assess a general trend, and we broadly conclude that the performance scores reported in this work hold true for all the class imbalance ranges covered for the datasets used.

### Can prior feature selection improve performance?

Models presented above have focused on generalizability across treatments and cancer types, and improved performance, utilizing most informative representations. ML-based models generally suffer from challenges due to tissue heterogeneity, sample biases, and the nature of feature data available, e.g. discussed in [40–42]. In this work, specifically, it is not clear at the outset if prior selection of features from the high-dimensional data will improve performance of these models. If an improvement in performance can be established by this procedure, it will be easier to also interpret the selected features, although that is not the focus of the current work. In order to evaluate the impact on the performance of the PCPT model, we adopted two methods of feature selection, namely (i) highly variable features and (ii) tensor decomposition-based unsupervised feature selection, as these two approaches have been shown to be powerful in improving model performance and interpretability in related problems [43, 44]. We applied both these strategies for selecting informative features, prior to applying PCA in the PCPT model. The rest of the methodology remains the same. The results of these experiments are discussed in the following.

#### Feature selection based on high variance

We performed high-variance filtering to retain the most variable features prior to applying PCA. From the complete omics dataset, we selected the 400 and 800 most variable features, which we label as the HVF-400a and HVF-800a feature set, respectively. We recall that our feature sets contain four modalities, namely CNA, GEX, CNUM, and SNV. However, we noticed that selecting features based on the highest variance retained only GEX features, because these features generally have a higher variance. To account for this, we also selected 100 highly variable features from each of the four modalities of features, yielding a total of 400 features. This feature set is labeled HVF-100e. In this way, we had the two models based on highly variable features, namely HVF-400a and HVF-800a, and HVF-100e, which was based on the 100 most variable features from each modality. As shown in Table 4, overall prediction performance based on these features did not show any significant performance over the PCPT model without feature selection.

**Table 4.** Prediction performance of PCPT models with prior feature selection using (A) highly variable features (HVF) and (B) tensor decomposition (TD)

		HVF-100e		HVF-400a		HVF-800a	
(A)	PCPT (mean)	(mean)	(*P*-value)	(mean)	(*P*-value)	(mean)	(*P*-value)
F1 score	0.5974	0.5694	0.2663	0.6311	0.1021	0.5809	0.5244
MCC	0.2982	0.2667	0.0892	0.3206	0.2577	0.3192	0.2240
Accuracy	0.6483	0.6120	0.0295	0.6484	0.9975	0.6355	0.4443
AUC of ROC	0.6060	0.5804	0.0302	0.6433	0.0023	0.6224	0.0922
		**TD-100e**		**TD-400a**		**TD-800a**	
(B)	**PCPT** (mean)	(mean)	(*P*-value)	(mean)	(*P*-value)	(mean)	(*P*-value)
F1 score	0.5974	0.6027	0.8140	0.5937	0.8699	0.6070	0.6864
MCC	0.2982	0.3156	0.3839	0.3130	0.4377	0.3356	0.0834
Accuracy	0.6483	0.6322	0.2761	0.6430	0.7053	0.6451	0.8387
AUC of ROC	0.6060	0.6247	0.1139	0.6241	0.1866	0.6247	0.1301

The column label suffix 100e with HVF and TD refers to 100 features selected from each of the four modalities, and 400a/800a refer to an overall 400/800 number of features selected ignoring their modality. PCPT model performances are repeated for quick comparison. *P*-values are computed using a paired Student's *t*-test.

#### Feature selection based on unsupervised tensor decomposition

We employed tensor decomposition-based unsupervised feature selection (TDbasedUFE) [43, 44] to identify the most informative features across four modalities in our data. The omics dataset was structured using the PrepareSummarizedExperimentTensorSquare function from the TDbasedUFE package (v1.8.0), which converts each omics data matrix into a squared format representing linear kernel relationships and bundles them into a comprehensive tensor architecture. We applied higher order singular value decomposition (HOSVD) to the constructed tensor using the computeHosvdSquare function to identify the latent factors that capture sample-to-sample relationships across omics types and cross-omics interaction. Optimal singular value vectors were then selected through the selectSingularValueVectorLarge function, guided by condition vectors incorporating sample labels (Sen or Res). Feature selection was performed using the selectFeatureSquare function by applying omics-specific standard deviation thresholds (CNA, 0.03; SNV, 0.03; GEX, 0.03; CNUM, 0.12) that were optimized through iterative analysis. This generated, for each omic type, a list of logical vectors indicating feature selection status and the raw *P*-value quantifying statistical significance. The final feature selection strategy integrated results across all four modalities using the tableFeaturesSquare function applied iteratively for each category. Features were ranked primarily by *P*-values derived from tensor decomposition analysis and secondarily by adjusted *P*-values following Benjamini–Hochberg multiple testing correction. From this comprehensive ranked list, encompassing all omics types, we systematically selected the top 400 and 800 features, which we labeled as TD-400a and TD-800a feature sets, respectively.

PCPT models were trained on these selected features and the results are shown in Table 4. We observe that although some performance metrics improved on average, the performance metrics have a high variation, resulting in statistically insignificance. Therefore, we conclude that improving performance of a PCPT model using feature selection in a robust manner is not straightforward and requires much more work and perhaps more PDX data which might become available in the future.

## Discussion

We have successfully developed an integrative genomics–transcriptome-based approach to predict treatment responses for multiple cancer types using PDX data. Even though performance levels are modest, they are not only comparable with the state-of-the-art models available for single cancer types but also provide a much greater coverage of patient groups. The results also suggest that the genomic profiles contain significant information about the specific responses to treatments by individual patients, of which we have been able to capture at least some. Combined with other clinical contexts, these results will be helpful in the personalized selection of cancer therapies with the aim of better treatment outcomes.

## Data Availability

All raw and processed data, along with analysis scripts, are available at: https://github.com/Sciwhylab/pcpt and https://doi.org/10.5281/zenodo.16605890.
